# Test method for de-icing salt-frost scaling in high-performance concrete

**DOI:** 10.1016/j.mex.2018.10.007

**Published:** 2018-10-09

**Authors:** Martin J. Strand, Katja Fridh

**Affiliations:** Division of Building Materials, Lund University, Lund, Sweden

**Keywords:** De-icing salt-frost scaling in high-performance concrete, Test method, High-performance concrete, Durability, De-icing salt-frost scaling, Freeze–thaw cycle

## Abstract

This paper describes a de-icing salt-frost scaling test method for analysis of the salt-frost scaling behaviour in high-performance concrete with various binders. The method was therefore designed to result in considerable scaling damage for the concrete considered to be salt-frost resistant. In addition, the experimental set-up was designed to avoid leakage, and to allow testing of a large number of samples. The method was validated by testing concrete with three different binders with a water–binder ratio of 0.40 with 5% air content. Various preconditioning procedures and freeze–thaw cycles were evaluated. The results show that the freeze–thaw cycle chosen results in a large mass of scaling and the salt-frost scaling behaviour agreed with the findings of previous studies. Thus, the method was considered suitable to study the salt-frost scaling behaviour in high-performance concrete. Three distinguishing features of the method are the following:

• The freeze–thaw cycle result in a large mass of salt-frost scaling to enable study of high-performance concrete.
• The concrete sample is above the salt solution to prevent leakage. The test surface is submerged 2 mm into the salt solution inside a cup.
• Freezers with air as the thermal medium are used to allow a large number of samples.

## Graphical abstract

**Figure:**
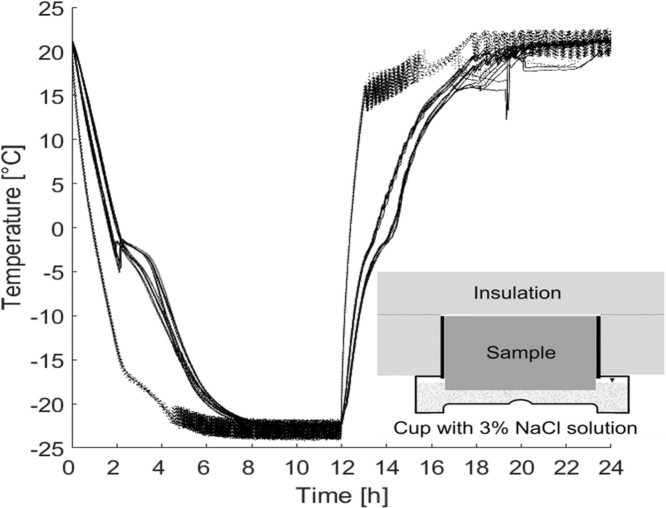

**Specifications Table**

**Table:** 

Subject area	• *Materials Science*
More specific subject area	This is a method testing the de-icing salt-frost scaling behaviour in high-performance concrete. De-icing salt-frost scaling is a type of superficial damage that occurs when a low concentration of de-icing salt solution freezes in contact with a concrete surface.
Method name	De-icing salt-frost scaling in high-performance concrete
Name and reference of original method	CEN/TS 12390-9 (2006) Testing hardened concrete - Part 9: Freeze–thaw resistance - Scaling. 24 p. Fagerlund G (1982) The influence of slag cement on the frost resistance of the hardened concrete vol 1. Swedish Cement and Concrete Research Institute, Stockholm Lindmark S (2010) On the Relation between Air void system parameters and Salt frost scaling. Nordic Miniseminar: Freeze–thaw testing of concrete, Vedbaek, Denmark. Nordic Concrete Federation, pp 41-58 Utgenannt P (2004) The influence of ageing on the salt-frost resistance of concrete. Doctoral Thesis, Faculty of Engineering, LTH, Lund University
Resource availability	Concrete mixer and related equipment for casting concrete. Air content tester for fresh concrete. Materials desired in the concrete. Cylinder moulds with 100-mm diameter. Concrete saw. Climate chamber with a stable temperature, relative humidity and carbon dioxide concentration. Logger able to record temperature, relative humidity, and carbon dioxide concentration. Freezer able to cycle between –25 °C and +25 °C. Materials specified in section ‘1. Method details’.

## Method details

De-icing salt-frost scaling is a type of superficial damage that occurs on concrete structures. This happens when water with a low concentration of de-icing salt freezes in contact with a concrete surface. The outermost layer of the concrete structure protects the reinforcement bars. Thus, if the concrete is not salt-frost scaling resistant, the protective concrete layer will scale off as a result of repeated freezing and thawing. This will in turn shorten the period of time until the reinforcement bars start to corrode; thus, the service life of the concrete structure is shortened. There are various methods to assess the salt-frost scaling resistance of concrete [[1], [2], [3], [4], [5], [6], [7]]. All of these methods use a low-concentration salt solution. This solution is in contact with the concrete test surface during the test. The salt solution and concrete are then exposed to a freeze–thaw cycle. The conditions of the freeze–thaw cycle are different (cycle duration, max and min temperature) for each salt-frost scaling test method.

Two important features of the proposed method are the freeze–thaw cycle and the sample setup. The method was designed to enable the testing of high-performance concrete, which generally has a high salt-frost scaling resistance. Hence, the selected freeze–thaw cycle should result in a large mass of scaled material. The method should also be able to detect how different preconditioning processes affect the salt-frost scaling behaviour with various binders. Since a large mass of scaled material can result in leakage of salt solution if the solution is on top of the surface, the test surface was instead submerged into a salt solution inside a cup. The sample size was chosen to allow a large number of samples to be tested at the same time.

### Sample preparation

The preparation and preconditioning of the test surface have a large influence on the salt-frost scaling results [6,[8], [9], [10], [11], [12]]. It is therefore essential that the preconditioning process is controlled and documented. Here, 200 mm long concrete cylinders with a diameter of 100 mm were cast. Each cylinder was later sawed into four concrete samples. Therefore each sample had a length of approximately 49 mm and a diameter of 100 mm. Only sawed surfaces were tested to minimise the influence of inhomogeneity at the cast surface which can increase the spread in the results.

**Fig. 1:**
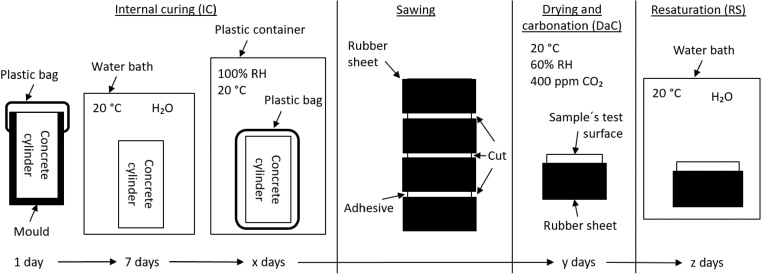
Overview of the preconditioning process and preparation of the concrete samples including internal curing (IC), sawing, drying and carbonation (DaC), and resaturation (RS).

The preconditioning of the test surfaces comprised three stages, as shown in Fig. 1, namely, internal curing (IC), drying and carbonation (DaC), and resaturation (RS). IC consisted of sealed hydration before the concrete cylinders were sawn. Here, the cylinders were cured in the moulds for 24 ± 2 h. After de-moulding, the cylinders were submerged in lime-saturated tap water for seven days and were then placed in plastic bags until they were sawn. DaC occurred after the concrete cylinders had been sawn. In this step, the concrete sample test surfaces were exposed to +20 ± 0.5 °C, 60 ± 2% relative humidity (RH) and about 400 ppmv CO_2_. During the RS period, the test surfaces were exposed to tap water before the start of the salt-frost scaling test.

The sides of each concrete sample were sealed with a rubber sheet (length: 330 ± 1 mm, width: 40 ± 1 mm). This was normally done during the DaC period, but if the preconditioning did not include a DaC period, this was done during the IC period. The rubber sheet was fastened with an adhesive. To enable the adhesive to attach to the surface, the concrete cylinders were taken out of the plastic bags for about 1 h to dry before the rubber sheet was fastened to the surface by the adhesive. Since the rubber sheet was only 40 mm wide, sections of the cylinder were left with only a layer of adhesive where the saw was applied (Fig. 1). This was done to avoid sawing into the rubber which would rip the rubber off. The sawn concrete samples were then placed back into the plastic bags. If the preconditioning did not include any DaC, the samples were placed directly into water after they had been sawn, for the RS period.

### Preparation of sample setup

**Fig. 2:**
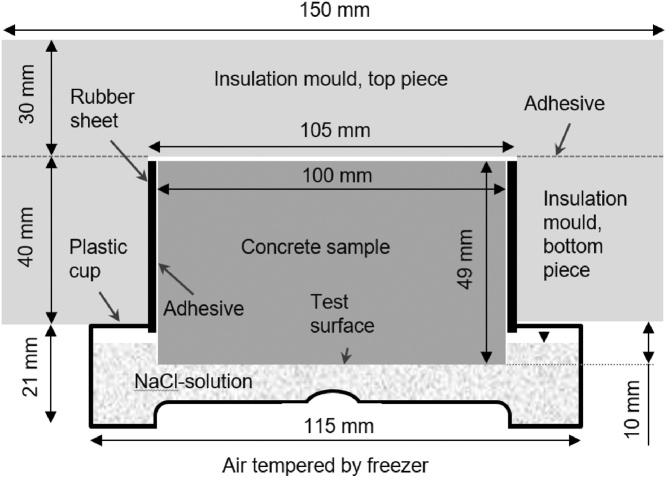
Section of the sample setup including the extruded polystyrene insulation mould (XES 300 j, Paroc AB, Skövde, Sweden), the EPDM-rubber sheet (3 mm 120 ± 20 kg/cm³, Lundgrens Sverige AB, Gothenburg, Sweden) fastened with a silyl-modified polymer adhesive (Xtreme fix, Casco, Sika Sverige AB, Spånga, Sweden), and a polypropylene plastic cup (S Unipac Square, Nordic Pack, Nykvarn, Sweden). The 3 mass% NaCl solution was deionised water and 99.9% NaCl (Falksalt, Gothenburg, Sweden).

The sample setup is presented in Fig. 2. The insulation mould was made by gluing two pieces of insulation together. Before the two pieces were fastened together, a hole (ø 105 mm) was drilled in the bottom piece. Since the elastic rubber sheet had a thickness of 3 mm, the total diameter of the sample, adhesive, and elastic rubber sheet was 106 mm. This meant that the sample could be pressed into the insulation mould. This eliminated the need for any spacers inside the cup for the sample to stand on.

The test surface was submerged 2 mm into the salt solution. To achieve this, the sample test surface should be 10 mm from the insulation mould. This was accomplished consistently by using a distance aid with a thickness of 10 mm (length: 150 mm, width: 150 mm) and a hole in the middle with a diameter of 120 mm. The concrete sample was placed inside the hole in the distance aid, with the test surface in contact with the table. The insulation mould was then pressed down on the sample until it was in contact with the distance aid.

Each cup was cut from the bottom of a plastic jar. The cup were then filled with 100 ± 1 ml of 3 mass% NaCl solutions using a 100 ml plastic syringe. The 3 mass% NaCl solution was made by mixing 4850 g deionised water and 150 g salt with NaCl. The insulation mould together with the concrete samples were then placed on top of the cup. This resulted in the sample setup shown in Fig. 2.

### Measurement procedure

**Fig. 3:**
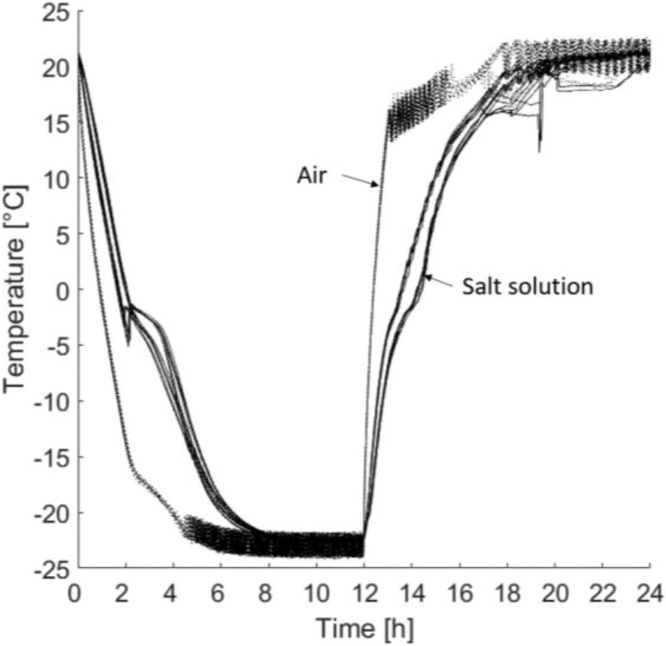
Temperature cycle measured in the air inside a freezer and in the salt solution in a cup.

The samples were placed inside a freezer (CF-A700, Kylcity, Skogås, Sweden). Each freezer had seven shelves and nine samples were placed on each shelf. The samples were then exposed to a pre-defined temperature cycle in which the air inside the freezers was maintained at −22 °C for 12 h and +22 °C for 12 h. Fig. 3 presents the temperature cycle, both in the salt solution and in air. It was important to always measure the temperatures inside the freezer and inside at least one cup in each freezer with a known position of the thermocouple. For additional information regarding the temperature cycle, see section ‘2.2 Methodical aspects – *Temperature cycle*’.

**Fig. 4:**
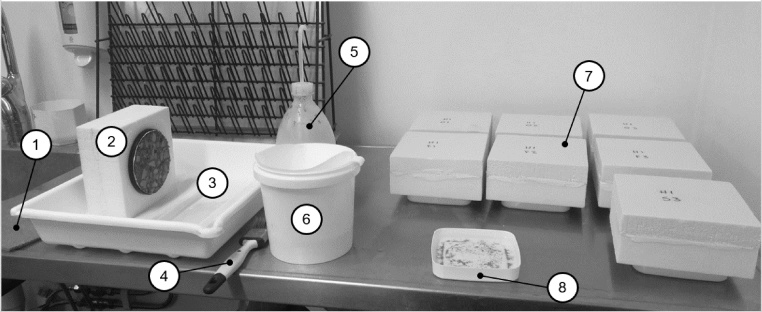
Equipment used during a measurement. ① Wet cellulose-based cloth (Wettex, Norrköping, Sweden), ② sample ready to be brushed, ③ open plastic tray for brushing test surface, ④ paint brush, ⑤ plastic flask with tap water, ⑥ plastic container with holder for filters and filter (Melitta Original 1 × 4 coffee filters (white), Melitta Unternehmensgruppe Bentz KG, Minden, Germany), ⑦ samples from the same shelf inside the freezer, and ⑧ plastic cup containing scaled material and salt solution.

Fig. 4 show all the equipment used during the measurements. The following measurement procedure was done every seventh cycle. First, paper filters were prepared, and a batch of salt solution was mixed. The filters were marked with the sample name and the number of freeze–thaw cycles that each sample had been exposed to. When new salt solution and filters had been prepared, one shelf at a time was taken out of the freezer. All the samples from the shelf were placed on a working bench in the same order as on the shelf (⑦ in Fig. 4). Each sample was gently removed from its plastic cup and was placed inside an open tray (②, ⑧ and ③ in Fig. 4). At that point, it was important to check that the test surface has been in contact with the salt solution. This was done by observing whether there was any notable movement in the salt solution during removal of each concrete sample. If this was not the case, some solution was assumed to have leaked from the cup, and this was noted. Then the salt solution and the scaled off material was poured into a paper filter (⑥ in Fig. 4). The test surface of each sample was brushed about 15 to 25 times in four perpendicular directions (fewer times when there was less mass scaling and vice versa) with a paint brush (④ in Fig. 4). Thereafter, the surface was rinsed with tap water which was collected in an open tray (⑤ and ③ in Fig. 4), and the sample was placed on a wet cellulose-based cloth (① in Fig. 4). All water on the test surface and the insulation mould was then wiped off. Then, the mass of each sample, including the insulation mould, rubber sheet, and adhesive was determined using a balance with 0.1 g resolution. The cup was then refilled with new salt solution, and the sample was placed back into the cup. The sample together with the plastic cup were then placed at the same position on the shelf as before. The scaled material that was collected in the tray was then poured into the filter as well. The filters containing the scaled material were then dried at 105 ± 5 °C until the change in mass was less than 0.001 g. The dry mass of the scaled off concrete was determined using a balance with 0.001 g resolution, and the mass of the filter was subtracted. Each step in the measuring procedure was designed with the aim to minimise spread in the results due to the method.

## Methodical aspects

### Choice of cups

With the proposed sample setup, the choice of material and size of the cup is important. Pre-tests were performed using petri dishes made of tempered glass, but they cracked during the measurements. A second test of cups was made using polypropylene plastic cups with the diameter of 115 mm. During this test, the solution sometimes leaked from the cups. This leaking was believed to be caused by insufficient volume of the cup. Instead, quadratic polypropylene plastic cups with the 115 mm sides were tested and did not leak. Thus, these cups were chosen to be used in the method.

### Temperature cycle

When designing the freeze–thaw cycle, the aim was to create a tough cycle that would yield a large mass of scaled material. The reason for this was to enable testing of high-performance concrete with a water binder ratio of 0.40 with 5% air content. The chosen temperature settings gave a cycle where the test surfaces were exposed to −22 °C for slightly more than four hours. Previous studies have shown that lowering the minimum temperature increases the mass of scaling [10,[13], [14], [15]]. Therefore, this cycle can be considered tough with regard to the minimum temperature. Another factor that makes the cycle tough is that an increased period of time when the samples are exposed to the minimum temperature increases the mass of scaled material [11]. An additional advantage of the temperature cycle is that it provides more than eight hours to perform the salt-frost scaling measurements after each freezing period. This is enough to finish measurements on 63 samples during one thawing period.

**Fig. 5:**
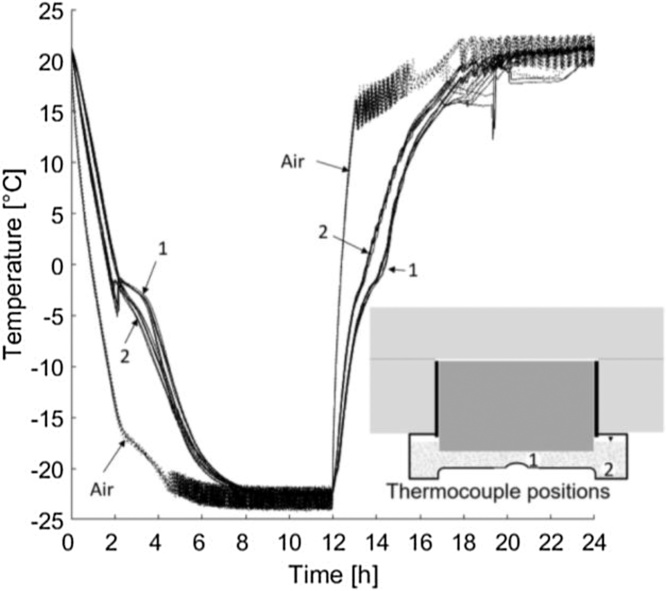
Temperature measurements from 14 freeze–thaw cycles. Seven measured cycles in position 1 and seven measured cycles in position 2 with the same thermocouple.

**Fig. 6:**
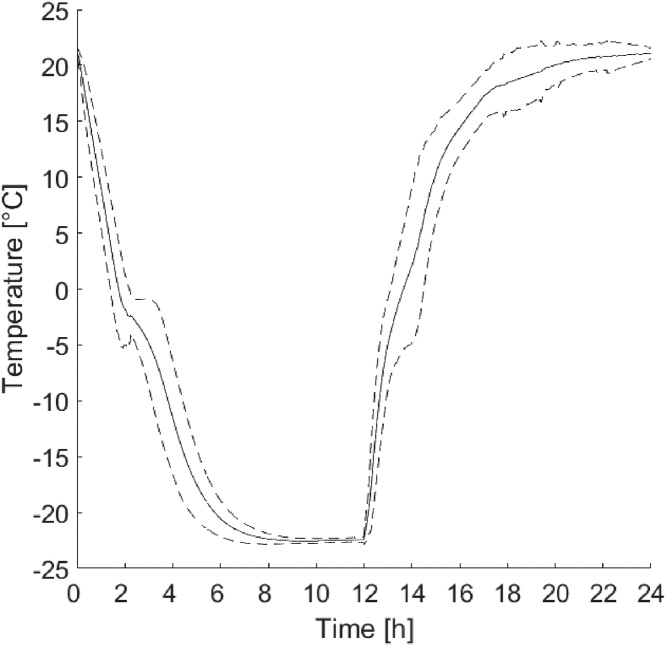
Mean value and mean value plus or minus two times the standard deviation of 98 cycles.

To investigate the importance of the position of the thermocouple, measurements were made inside in the solution of seven samples during 14 cycles each. During seven of these cycles the thermocouple was in position 1, shown in Fig. 5, and in position 2 for the other seven. The difference in temperature between the two positions was investigated because the bottom of the plastic cup was not flat. However, the position of the thermocouple had no significant impact on the temperature. In Fig. 6, measurements from 14 cycles for seven different samples inside the solution are presented. Seven cycles was measured in each of the two positions. These seven samples were on seven different shelves during the measurements. These measurements show that the setup gives a low spread in temperature during the cycle despite the different locations of the thermocouple.

Tests were made to optimise the positioning of the samples inside the freezer. This was done to obtain a low spread in the temperature cycle for each sample and to maximise the number of samples that could be placed in the freezer. This was done by varying the number of shelves and the number of samples on each shelf. However, the air circulation inside the freezers gave an even temperature cycle for all samples. Thus, the number of samples that could be tested in the freezer simultaneously was only limited by the available space.

## Method validation

The method was assessed to confirm that it can differentiate between concrete with three different binders preconditioned in different ways. Three different factors were studied: the freeze–thaw cycle, the DaC period, and the RS period. The basic recipes are presented in Table 1 and the preconditioning process of each cast is presented in Table 2. Comparisons between the results are made of samples with the same total hydration period without external drying of the test surface, i.e. IC plus RS according to Table 2. The hydration of the test surface during the DaC period is assumed to be negligible. Therefore, comparing samples with the same binder that had the same total period of IC plus RS, it is considered a fair comparison with regards to the degree of hydration. One cast was made with each recipe for each of the three tests. More information about the materials can be found in ‘Additional information’.

**Table 1:** Basic recipes for the materials used in testing of the method.

Binder	CEM I	Fly ash	Slag	Water	Aggregates		Air content
	[kg/m³]	[kg/m³]	[kg/m³]	[kg/m³]	0–8 [kg/m³]	8–16 [kg/m³]	[%]
100% CEM I	430.0	0	0	172	925.5	786.7	ca. 5
65% CEM I + 35% Fly ash	279.5	150.5	0	172	925.5	786.7	ca. 5
65% CEM I + 35% Slag	279.5	0	150.5	172	925.5	786.7	ca. 5

**Table 2:** Preconditioning of the samples used for each test during the validation of the method.

Factor tested	Internal curing (IC) [days]	Drying and carbonation (DaC) [days]	Resaturation (RS) [days]	Internal curing + resaturation (IC + RS) [days]
Shortened freezing period Prolonged freezing period	36 64	0 0	1 1	37 65
DaC period	30 30	0 87	1 1	31 31
RS period	28 55 56	31 31 32	28 1 0	56 56 56

### Freeze–thaw cycles

**Fig. 7:**
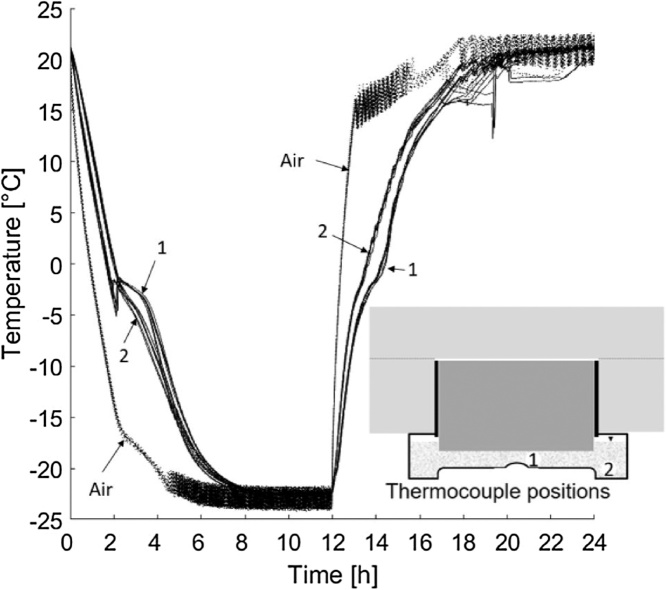
Freeze–thaw cycles studied; data from [16]. The graph shows two measurements for each cycle. All measurements were made inside the salt solution with the thermocouple at position 1 according to Fig. 4.

**Fig. 8:**
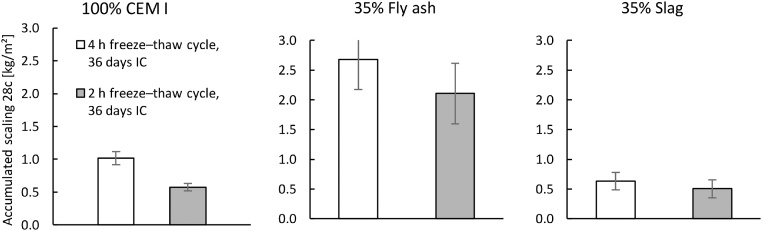
Influence of a shortened freezing period (cycles ‘4 h’ and ‘2 h’ in Fig. 6) on the salt-frost scaling for each binder. All samples had 36 days of IC and one day of RS (no DaC period). Mean accumulated salt-frost scaling and standard deviation for six samples (from cast #3 of each binder) after 28 cycles; data from [16].

**Fig. 9:**
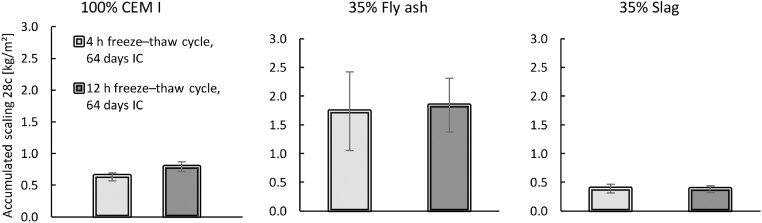
Influence of a prolonged freezing period (cycles ‘4 h’ and ‘12 h’ in Fig. 6) on the salt-frost scaling for each binder. All samples had 64 days of IC and one day of RS (no DaC period). Mean accumulated salt-frost scaling and standard deviation for six samples (from cast #3 of each binder) after 28 cycles; data from [16].

**Fig. 10:**
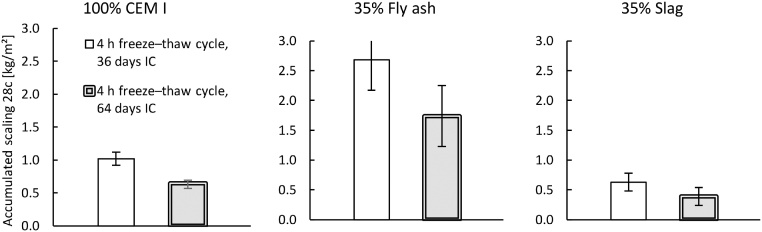
Influence of an increased period of IC when samples were exposed to a 4-h cycle on the salt-frost scaling for each binder. Mean accumulated salt-frost scaling and standard deviation for six samples (from cast #3 of each binder) after 28 cycles; data from [16].

The first validation test studied the effect of different freeze–thaw cycles, with three variations of the freezing and thawing times, as shown in Fig. 7. The times each sample was exposed to temperatures below −21 °C in each cycle were approximately 2 h, 4 h, and 12 h. All cycles were 24 h in total. The results are presented in Fig. 8, Fig. 9. For a comparison of the 2-h and 4-h cycles, the samples were preconditioned in the same way (‘Shortened freezing period’ in Table 2). The samples used to compare the 4-h and 12-h cycles were also preconditioned in the same way (‘Prolonged freezing period’ in Table 2). In addition, a comparison was made between the two different preconditioning processes with samples exposed to the 4-h cycle as shown in Fig. 10.

The results presented in Fig. 8, Fig. 9 indicate that the salt-frost scaling increases when the freezing time is prolonged and the thawing time is shortened. These results agree with the findings of previous research [11,15]. As seen in Fig. 10, a comparison of the samples exposed to the 4-h cycle with either 36 days or 64 days of IC, the scaling decreased with increased hydration time. These results also agree with the findings of previous research [6,[17], [18], [19], [20]]. Therefore, the method can evaluate the effect of different freeze–thaw cycles as well as the effect of increased hydration time on concrete with various binders.

### Drying and carbonation

**Fig. 11:**
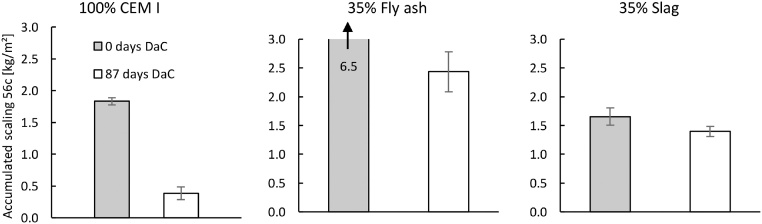
Influence of two different DaC periods on the salt-frost scaling for each binder. All samples were subjected to IC for 30 days and had one day of RS, in addition to the DaC period. Mean accumulated salt-frost scaling and standard deviation for six samples (from cast #1 of each binder) after 56 cycles.

The second validation test studied the effect of the DaC period by comparing samples that were exposed to 87 days of DaC (at 20 °C with 60% RH and about 400 ppmv CO_2_) with samples exposed to zero days of DaC. Since water evaporates during the DaC period, the samples exposed to DaC contained a lower water content before the salt-frost test began. In addition, the surface of the samples exposed to DaC were carbonated before the salt-frost scaling test began. The samples that were not exposed to any DaC period were assumed to have had an uncarbonated surface and a higher water content after the RS period. The accumulated mass of scaling is presented in Fig. 11.

DaC resulted in a lower mass of salt-frost scaling for concrete containing 100% CEM I, which also was found by [6]. DaC also decreased the salt-frost scaling for concrete containing 35 mass% fly ash, but the samples still showed a large mass of salt-frost scaling. The concrete containing slag showed a very small decrease in the mass of salt-frost scaling from drying and carbonation. These results indicate that the method can distinguish the differences in the salt-frost scaling behaviour for the three different binders preconditioned with and without a DaC period.

### Resaturation

**Fig. 12:**
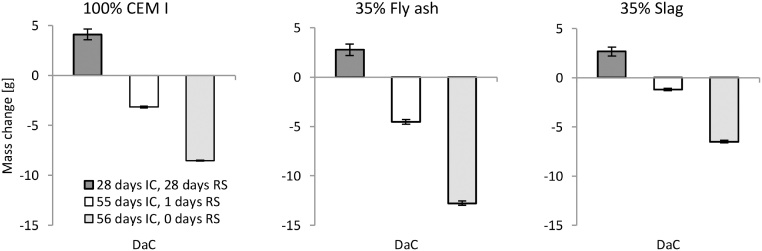
Mean mass change for three samples (from cast #2 of each binder) during the DaC and various RS periods along with the total mass change from the beginning of the DaC begins until the end of the RS period.

**Fig. 13:**
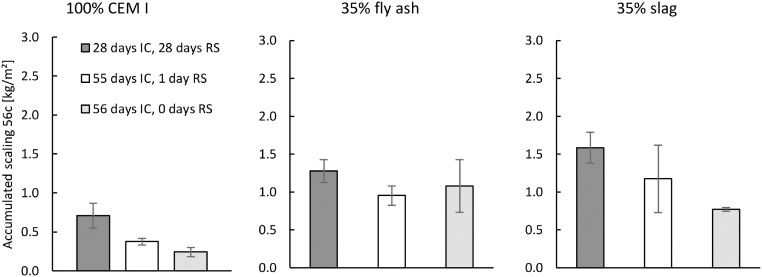
Influence of the RS period on the salt-frost scaling for each binder. All samples had 32 or 31 days of DaC in addition to IC and RS. Mean accumulated salt-frost scaling and standard deviation for three samples (from cast #2 of each binder) after 56 cycles.

The third validation test studied the effect of changing the RS period through a comparison of samples with three different RS periods, namely, 0 days, 1 day, and 28 days. Therefore, the main factor that was varied was the water content of the samples at the start of the salt-frost test. Fig. 12 presents the total mass change from the start of the DaC period until the end of the RS period, before the start of the scaling test. The IC was varied to have the same total period of IC and RS for all the samples to decrease the effect of different degrees of hydration when the salt-frost test began. The samples with 0 days RS were placed directly into the salt solution 2 ± 1 h before the first freezing cycle began. All of these samples were dried and carbonated for approximately 32 days before the RS period. The salt-frost scaling results are presented in Fig. 13.

DaC results in a reduction in mass due to evaporation of moisture close to the test surface, and RS results in an increase in mass due to water uptake. As seen in Fig. 12, the mass of samples exposed to 28 days of RS increased from the start of the DaC period until after the RS period. The samples exposed to 1 and 0 days of RS showed a decrease in mass, where samples with 0 days of RS had the lowest water content at the start of the salt-frost test.

The salt-frost scaling results are presented in Fig. 13. An increased RS period increased the mass of salt-frost scaling for concrete with 100% CEM I and concrete containing 35% slag. The results from concrete containing 35% fly ash are not as clear, but [10] also found the moisture history of the test surface is an important factor of the salt-frost scaling damage. Again, the method is able to distinguish the different behaviours of different binders preconditioned in different ways.

## Conclusions

• The sample setup and temperature cycles gave a tough temperature cycle with a small spread. This resulted in a large mass of scaling and enabled testing of high-performance concrete (water binder ratio of 0.40 with 5% air content).
• The method detects differences in de-icing salt-frost scaling behaviour for various factors, such as different binders, preconditioning processes, and freeze–thaw cycles.
• The method allows for a large number of samples to be tested simultaneously.
