# Supplementary material for: Test method for de-icing salt-frost scaling in high-performance concrete
Source: MethodsX. 2018 Oct 9;5:1299–310. doi: 10.1016/j.mex.2018.10.007 (PMC6199811; doi:10.1016/j.mex.2018.10.007)
Supplement: Supplementary file 1 [file mmc1.docx]

**Additional information**

| Cast | Slump | Density | Air Content | Air Content |
| --- | --- | --- | --- | --- |
|  |  |  | in concrete | in paste |
|  | [mm] | [kg/m³]^1)^ | [%]^1)^ | [%]^2)^ |
| CEM I - 1 | 210 | 2351 | 4.8 | 13.0 |
| CEM I - 2 | 60 | 2347 | 4.9 | 13.8 |
| CEM I - 3 | 100 | 2364 | 5.0 | 14.0 |
| F35 - 1 | 240 | 2266 | 5.5 | 14.6 |
| F35 - 2 | 30 | 2283 | 5.7 | 15.7 |
| F35 - 3 | 200 | 2369 | 3.8 | 11.0 |
| S35 - 1 | 230 | 2280 | 5.6 | 14.8 |
| S35 - 2 | 60 | 2307 | 4.9 | 13.8 |
| S35 - 3 | 190 | 2385 | 4.1 | 11.8 |

^1)^ Measured in 8 dm³ of the fresh concrete.

^2)^ Calculated based on the measured air content and compact densities of the binders and aggregates.

The cement used was CEM I 42,5 N - SR 3 LA (Cementa Heidelberg cement group, Stockholm, Sweden). The fly ash had a low calcium content (Emineral a/s, Fredericia, Denmark). The slag was a ground granulated blast-furnace slag (Merit 5000, Merox (part of SSAB), Stockholm, Sweden).

***Test of effects of DaC***

Mean accumulated salt-frost scaling after a certain number of cycles. The *y*-axis shows the accumulated salt-frost scaling divided by the total area of the samples after a certain number of cycles.

Coefficient of variation for accumulated scaling curves. The coefficient of variation is calculated as the standard deviation of the accumulated salt-frost scaling divided by the mean accumulated salt-frost scaling.

Mean change in mass during the DaC period (dehydration leads to a reduction in mass) where the conditions were 20 °C, 60% RH, and 400 ppm CO_2_.

***Test of various RS periods***

Mean accumulated salt-frost scaling after a certain number of cycles. The *y*-axis shows the accumulated salt-frost scaling divided by the total area of the samples after a certain number of cycles.

Coefficient of variation for accumulated scaling curves. The coefficient of variation is calculated as the standard deviation of the accumulated salt-frost scaling divided by the mean accumulated salt-frost scaling.

Mean change in mass during the DaC period (dehydration leads to a reduction in mass) where the conditions were 20 °C, 60% RH, and 400 ppm CO_2_ and the RS period (water absorption leads to an increase in mass) before the salt-frost scaling test began.

***Test of various freeze-thaw cycles***

Mean accumulated salt-frost scaling after a certain number of cycles for samples exposed to different cycles (FTC = freeze–thaw cycle). The *y*-axis shows the accumulated salt-frost scaling divided by the total area of the samples after a certain number of cycles.

Coefficient of variation for accumulated scaling curves. The coefficient of variation was calculated as the standard deviation of the accumulated salt-frost scaling divided by the mean accumulated salt-frost scaling.
